# Bending effect on the resistive switching behavior of a NiO/TiO_2_ p–n heterojunction†

**DOI:** 10.1039/c8ra01180j

**Published:** 2018-05-30

**Authors:** Hai-peng Cui, Jian-chang Li, Hai-lin Yuan

**Affiliations:** Vacuum and Fluid Engineering Research Center, School of Mechanical Engineering & Automation, Northeastern University Shenyang 110819 PR China jcli@mail.neu.edu.cn +86 13804075191

## Abstract

Herein, NiO/TiO_2_ heterojunctions were fabricated by sol–gel spin coating on plastic substrates to investigate the effects of bending on resistive switching. The switching mechanism is well explained by the formation and rupture of oxygen-vacancy conducting filaments modulated by the p–n interface. Compared with that of the unbent film, the device ON/OFF ratio is slightly improved after 5000 bending repetitions. Finite element calculations indicate that the tensile stress of 0.79% can lead to the formation of channel cracks. Further charge transport analysis shows that the conducting filaments may cause an incomplete rupture because the bending-induced channel crack permeates through the p–n interface and reduces the local depletion-region width.

## Abstract 2

The bending-induced micro-crack leads to the incomplete rupture of the conducting filaments and reduces the local depletion-region width.

## Graphical abstract

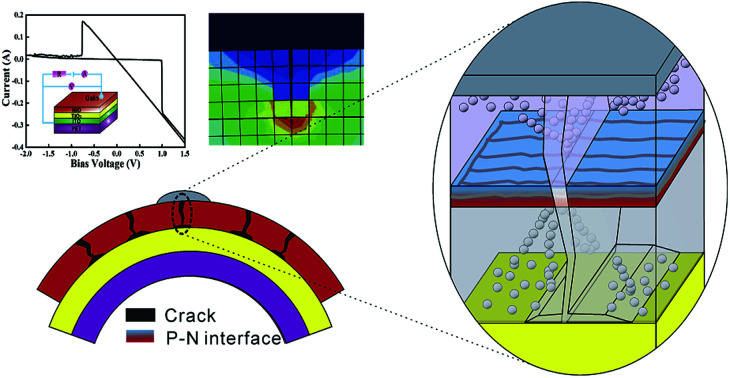

## Introduction

Resistive random access memory (RRAM) has attracted significant attention due to its high density, simple structure, facile processing, and fast switching.^1^ Various insulating or semiconducting materials, including graphene oxide,^2,3^ organic materials,^4^ and inorganic oxides (such as NiO,^5,6^ TiO_2_,^7^ ZnO,^8,9^ and TbMnO_3_ (ref. 10)), have been studied for RRAM applications. Among them, NiO as a p-type binary metal oxide has excellent physical and transport properties.^1^

To improve the resistive switching (RS) performance, the p–n junction is a key structure for most semiconductor devices.^11^ For example, Kim *et al.* fabricated an n-TiO_2_/p-NiO heterojunction and found that the formation and rupture of filamentary paths could be controlled at a special location by engineering the heterostructures.^12^ Zhou *et al.* observed the migration of oxide vacancies and charge trapping and de-trapping near the electrodes, which co-contributed to the enhanced RS memory phenomenon in the TiO_2_/NiO bilayer structure, and the oxygen-generated vacancy played an important role in the resistance switching memory effect.^13,14^ Chen *et al.* showed that the resistive behavior in the C_60_/MoS_2_ p–n nanojunction was due to electron tunneling *via* p–n junction barriers modulated by electric-field-induced polarization.^15^ However, how the p–n interface affects the switching under mechanical bending conditions is still under debate.

In the case of flexible electronics, resistive switching for Au/ZnO NR/Au memory devices was maintained under severe substrate bending.^16^ Similarly, Lee *et al.* noted a nearly constant threshold voltage and ON/OFF ratio for Mg/Ag-doped chitosan/Mg devices bent with a 5 mm radius of curvature under 1000 cycles.^17^ The resistance ratio of Al/ZnO nanorod-incorporated graphene oxide/ITO/PET devices was stable even after 10^3^ times bending with a 4 mm radius.^3^ Shang *et al.* studied an all-oxide ITO/HfO_*x*_/ITO structure fabricated on the polyethylene terephthalate (PET) substrate and found that the resistivity increased significantly with a decrease in the bending ratio and the cracks formed along the perpendicular direction when the strain value was over 2.12%.^18^ However, how the bending affects the resistive switching of p–n heterojunctions is still rarely explored. In this study, the NiO/TiO_2_ heterojunctions are spin-coated on ITO/PET substrates to fabricate devices with the GaIn/NiO/TiO_2_/ITO/PET structure. Moreover, bending-degree-dependent switching properties have been studied to learn the effect of bending on the RS of the films.

## Experimental

All the chemical reagents were of analytical grade and used without further purification. Ethylene glycol monomethyl ether, nickel acetate tetrahydrate [Ni(CH_3_COOH)_2_·4H_2_O], and equal molar amounts of diethanolamine were used as the solvent, precursor, and sol stabilizer for NiO, respectively. For the TiO_2_ sol, 15 ml tetrabutyl titanate was dissolved in 60 ml pure ethanol and named solution A, and a mixed solution with 3 ml aqua fortis and 15 ml pure ethanol was named solution B. Then, solution A was slowly added to solution B under stirring for 1 h. Details of the sol–gel synthesis for NiO and TiO_2_ nanoparticles have been previously described.^19,20^ The NiO and TiO_2_ films were successively deposited by spin coating on the Si (100) and ITO/PET substrate at 800 rpm for 5 s immediately followed by 3000 rpm for 30 s. Each layer with a thickness of about 200 nm was preheated at 100 °C for 5 min to remove the solvent and organic residuals. The GaIn liquid droplet was employed as the top electrode in our device. The bias voltage sweep sequence is 0 V → 3 V → 0 V → −3 V → 0 V with a relative humidity of 20%. Although TiO_2_ is very sensitive to the moisture level, which will influence the RS character,^21^ the moisture is constant in our case. The RS behavior of the films was tested using an LK-2005Z potentiostat in the bias sweeping mode under ambient conditions. The X-ray diffraction (XRD) patterns were obtained using an X'Pert Pro diffractometer.

The thickness of the bilayer film was obtained by examining a scanning electron microscopy (SEM) image of a cross-sectional specimen using a Shimadzu SSX-550 equipment. A robust simulation-based extended finite element method was performed using the commercial software ABAQUS.

## Results and discussion

**Fig. 1:**
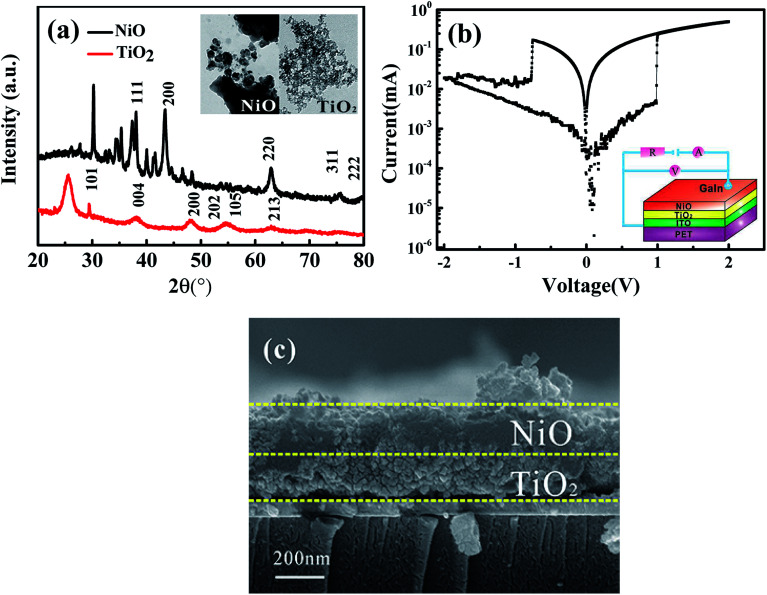
(a) Typical XRD patterns of the NiO and TiO_2_ thin films. The inset shows the TEM images of the as-grown NiO and TiO_2_ nanoparticles. (b) *I*–*V* characteristics of the GaIn/NiO/TiO_2_/ITO structure. (c) Cross-sectional SEM images of the sample.

Fig. 1(a) shows the XRD spectra of the NiO and TiO_2_ nanoparticle. The cubic phases of NiO and anatase TiO_2_ were well retained in the individual layers (JCPDS 01-075-0197 for NiO and JCPDS 01-078-2486 for TiO_2_). The nanoparticle sizes of NiO and TiO_2_ are around 24.5 and 4.1 nm, respectively, according to the Debye–Scherrer formula (see the inset of Fig. 1(a)). This is consistent with the TEM observation. The schematic configuration and electrical measurements of the heterojunction devices are depicted in Fig. 1(b), and the cross-section SEM image is shown in Fig. 1(c). As can be seen, the thicknesses of both the NiO and TiO_2_ films are about 200 nm.

As shown in Fig. 1(b), the NiO/TiO_2_ p–n heterostructure device presents bipolar switching without any metal filament formation. The form-free behavior can be attributed to the initial connection of tiny conducting filaments. Wang *et al.* previously observed a similar phenomenon while studying the InGaZnO film.^22^ As shown in Fig. 1(b), the device initially maintains a high resistance state (HRS) under forward bias from 0 to 1 V (the bias of 1 V is named as the SET voltage). When the bias reaches or exceeds the SET value, the current jumps abruptly to a high value; this indicates that the device switches from the HRS to a low resistance state (LRS). Subsequently, a RESET process is observed at −0.8 V during the negative voltage sweeping from 0 to −2 V.

**Fig. 2:**
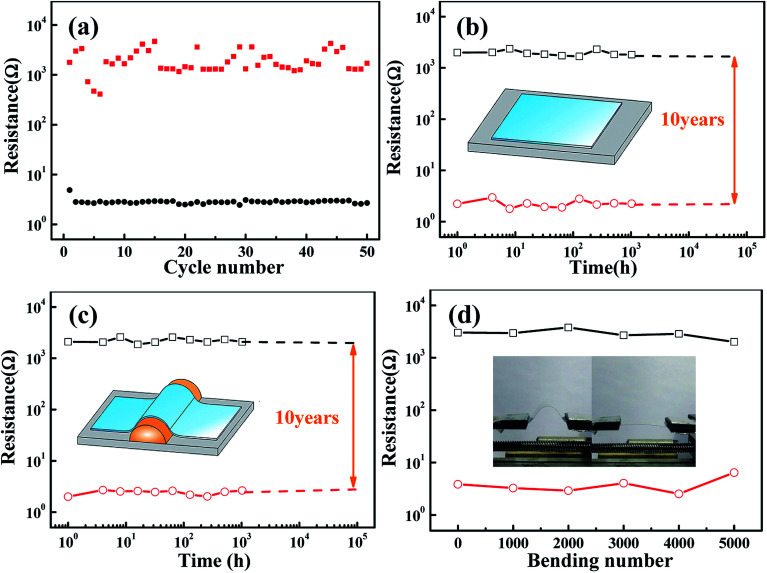
(a) Endurance performance of the NiO/TiO_2_ device in 50 successive bias scanning cycles. Retention test of the device under (b) unbending and (c) tensile strain conditions. (d) Mechanical stability test of the device with a bending radius of 10 mm.

The reproducibility and reliability of the NiO/TiO_2_ devices were investigated to evaluate the durability and retention properties of these devices. Fig. 2(a) shows the endurance characteristics at a reading voltage of 0.1 V obtained by repetitive DC sweeping,^2,23^ and the ON/OFF ratio is maintained at 10^2^ to 10^3^. During 50 endurance cycles, the resistances of HRS fluctuate to a certain extent, whereas those of the LRS are stable. On the basis of the filament model,^24–26^ the resistance of LRS is determined by the number and/or the size of conducting filaments.^27^ Since the size of the filaments in our sample is confined to the top electrode of the GaIn liquid droplet,^28^ the confinement effects on the filaments may result in relatively low values of the current for RS. Yan *et al.* showed that switching between different resistance states is usually abrupt, and the fluctuations of HRS are due to the random formation of filaments.^29^ Kumar *et al.* found that the formation of the conductive path was due to the high piezoelectric properties that reduced the hopping path for oxygen diffusion.^30^ A retention test was conducted to evaluate the data storage ability. As depicted in Fig. 2(b), the HRS and LRS can be retained for at least 10^3^ h without any significant degradation. The ON/OFF ratio only decreases by 11.1% after ten years, as indicated by the extrapolation.

To investigate the mechanical endurance and flexibility of our devices, bending tests were performed by direct probing on the bent sample with curvature radii of 10 mm (see Fig. 2(c)). It is shown that the decrease in the ON/OFF ratio is slightly higher as compared to that under flat condition. Furthermore, a repetitive bending test was carried out using a mechanical vibrator at a frequency of 1 bend/s. As shown in Fig. 2(d), the device shows an excellent endurance performance after 5000 bending cycles; this suggests its potential application in flexible nonvolatile memory devices. Interestingly, the ON/OFF ratio stabilizes at above 10^3^, which is higher than that under flat conditions (10^2^ to 10^3^). Wang *et al.* studied the Cu/α-IGZO/Cu structure^22^ and believed that this behavior might be due to the oxidation of oxygen-deficient α-IGZO buffer layer. In our case, the bending-induced heating effect and micro-cracks may accelerate the oxidation process in the device.^19^

The electrical performance of a flexible memory device is correlated to the evolution of cracks. Chang *et al.* have reported that oxygen or metal ions in metallic oxides are easily diffused and accumulate at the grain boundary.^31^ The tensile strain can affect the states of grain boundaries and influence the resistive switching of the heterostructure.^32^ Guan *et al.* have indicated that cracks may form in oxides at relatively low mechanical strains due to their brittle nature.^33^ For the case of crack formation in the function layer, the resistance may increase upto a specific level and then lead to the initiation of the final failure.^33,34^ Chung *et al.* have shown that the resistive switching of the Al/GeO_2_ : S/Au device can withstand a tensile or compressive strain of up to 1.2%.^35^ Shang *et al.* have reported that cracks appear in the ITO/HfO_*x*_/ITO film coated on the PET substrate when the mechanical tensile stress is up to 1.59%.^20^ In our case, the strain applied to the substrate is 0.79%, as calculated by the following equation:1
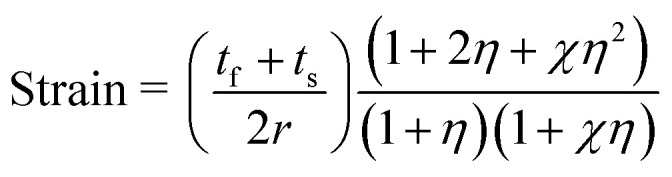
where *r* is the bending curvature radius of the PET substrates, *t*_f_ and *t*_s_ are the thickness of the total film and the substrate, respectively, *η* = *t*_f_/*t*_s_, *χ* = *E*_f_/*E*_s_, and *E*_f_ and *E*_s_ are the Young's moduli. The related film thicknesses and material properties are listed in Table 1. At the strain level of 0.79% in our case, the device may not reach the critical strain. Similarly, Lee *et al.* have found that the switching performance of the Au/ZnO/stainless steel structure is even improved after bending.^32^

**Table 1:** Material parameters of flexible composite films utilized in the FEA

Material	Young's modulus	Poisson's ratio	Thickness
ITO	118	0.3	80 nm
PET	2.9	0.37	175 μm
NiO	100	0.21	200 nm
TiO_2_	89	0.27	200 nm

**Fig. 3:**
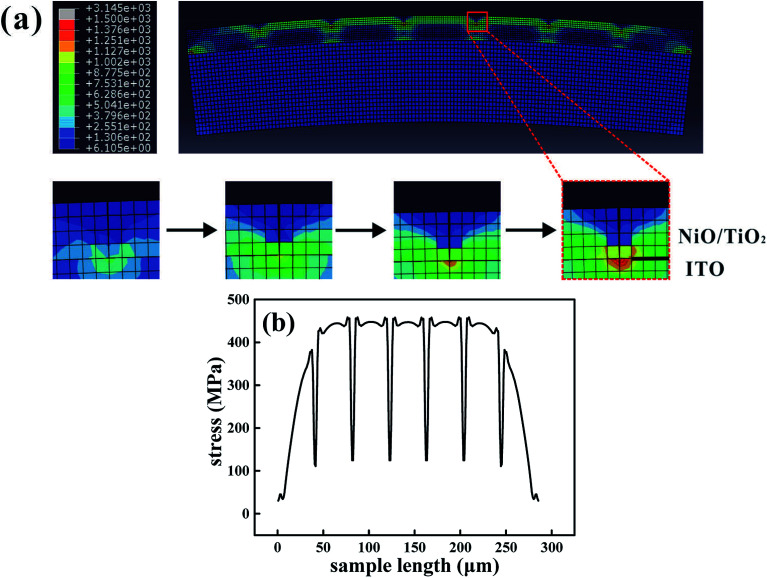
(a) Finite element analysis of the NiO/TiO_2_ heterojunction under the bending condition. (b) Stress distribution through-length direction with a 10 mm bending radius.

The finite element analysis (FEA) was performed using the commercial software ABAQUS to understand the bending effects on the resistive switching. When the device is bent convexly, the flexural strains can be approximately regarded as tensile stress. Considering a large-sized thin-stacked film configuration and the large mismatch between the function layer and the PET substrate, a 2D model based on a plane strain condition is established. We speculate perfect bonding between the substrate and the layers, and the initial defects are uniformly distributed. The stress contour under bending is shown in Fig. 3(a); a qualitative description indicates that a stress concentration is more easily encountered around the crack. The predicted magnitude of flexural stress in each film is examined by the relatively larger Young's modulus. The function layer is divided into about 300 points, and the stresses are plotted in Fig. 3(b). A dramatic stress relaxation indicates that a group of parallel cracks in the NiO/TiO_2_ layer begins to initiate and propagate at a bending radius of about 10 mm. The channel crack can easily form in grain boundaries,^36,37^ and the diffusion paths of oxygen vacancies or metal ions are thus limited. As a result, the resistances in the HRS are slightly improved after bending. Moreover, the bending-induced heating effect accelerates the oxidation process and reduces the density of oxygen vacancy in the NiO/TiO_2_ layer.^38^

**Fig. 4:**
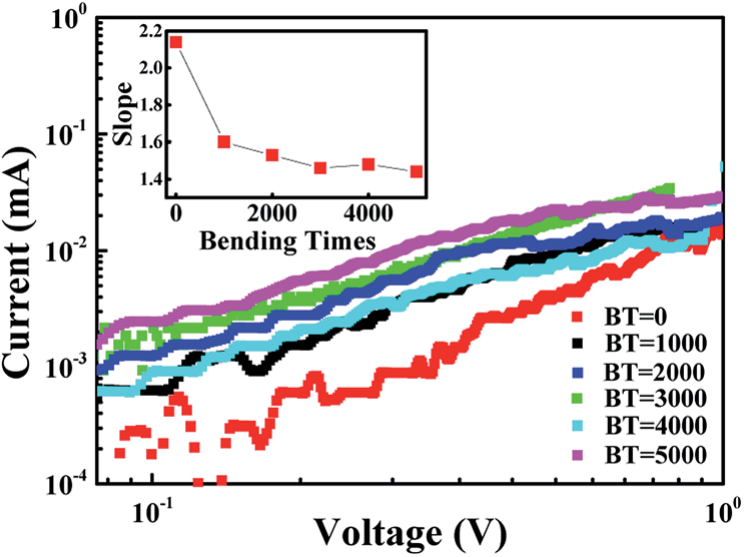
Logarithmic plot and shop for HRS of *I*–*V* curves with different bending times (BT).

To understand the bending effects on the charge transport of the NiO/TiO_2_ films, the *I*–*V* curves are analyzed in terms of the available theoretical models^23^*i.e.*, Schottky emission, space-charge limited conduction (SCLC), and Poole–Frenkel emission.^39^ The best fit originates from the SCLC both before and after bending.^25,40^ For the LRS, all the samples follow the ohmic law (see the ESI†).^3,32^ As shown in Fig. 4, the slope of *I*–*V* curve in HRS gradually decreased from 2.14 to 1.44 with an increase in bending times to 5000. The relatively lower value (2 in theoretical) under bending conditions indicates that the current conduction cannot be simply explained by an electron trapping and detrapping process, as found in other oxide systems.^2^ As discussed later, this phenomenon can be attributed to the penetration of micro-cracks through the p–n interface leading to the incomplete rupture of the conducting filaments.

**Fig. 5:**
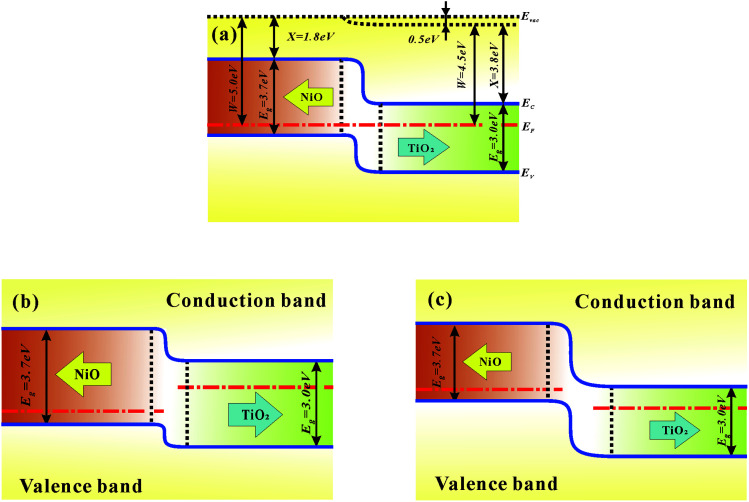
Energy band diagrams for NiO/TiO_2_ in a (a) dynamic equilibrium, (b) forward bias, and (c) reverse bias.

In conjunction with both the experimental and theoretical evidence, the resistive switching mechanism can be attributed to the cooperation of the interface and filament effect.^41^ Usually, metal filament formation is due to the diffusion of the top metal electrode.^42^ However, there is no metal atom deposition involved in our NiO/TiO_2_ layer, and the possibility of metal filament formation is thus ruled out. The formation of oxygen-vacancy filaments should be the main mechanism in the set process. According to previous studies, the filament rupture strongly depends on the p–n junction.^20^ Initially, the films are in the HRS, the p–n junction with a certain depletion width after reaching dynamic equilibrium (see Fig. 5(a)). Upon setting the applied electric field to a relatively low value, the injected electrons will gradually accumulate on the NiO side. The oxygen vacancies are aligned along the grain boundaries on the TiO_2_ side.^43^ The steadily increased electron density in the p–n junction reduces the depletion-region width (see Fig. 5(b)); this allows electrons to tunnel through the narrower barrier, and the conductive filaments are thus able to pass through the p–n junction to set the device to the LRS. The filaments are stable and nonvolatile until a reversely applied electric field is high enough to lead the barrier back. Once the accumulated oxygen vacancies and electron at the p–n interface are relaxed to a certain level, the barrier height will resume the initial height; this will cut off the conductive filaments to reset the device to HRS.

**Fig. 6:**
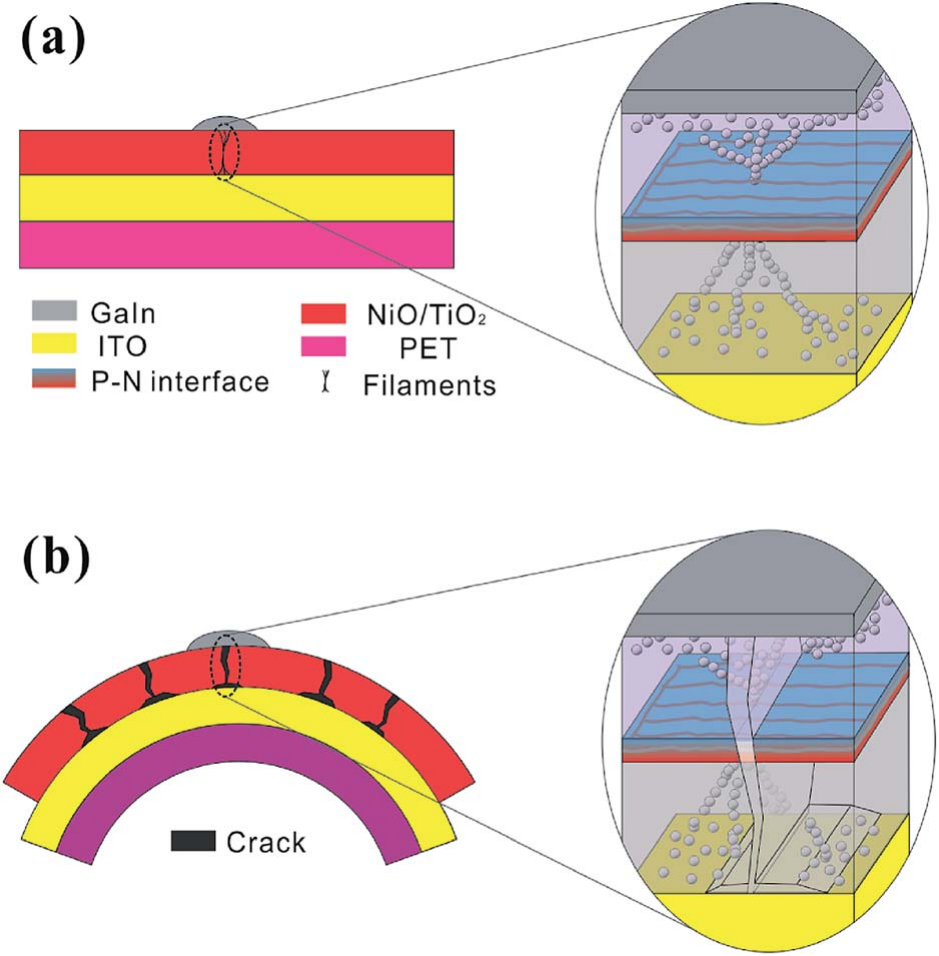
Schematic of the NiO/TiO_2_ device under (a) flat and (b) bending conditions.

To reveal the failure mechanism, the flexible memory device is schematically modeled under both flat and bent conditions (see Fig. 6). As stated above, the oxygen vacancy filaments connect the top and bottom electrodes under a flat stage, with branched filament dendrites distributed along the grain boundaries (see Fig. 6(a)).^44^ When the device is bent, cracks will form on grain boundaries along the direction perpendicular to that of the tensile strain.^18^ The crack can be thus considered as parallel to the filaments, which will cut off the branch of the filaments (see Fig. 6(b)) and increase the HRS resistance.^22^ Moreover, oxygen in air can easily permeate through the films along the micro-crack and accelerate the oxidation process in the p–n interface as well as reduce the local depletion-region width. The abovementioned analysis further confirms that the bending-induced micro-crack is responsible for the incomplete rupture of the conducting filaments. These explanations are well consistent with the experimental observation about the smaller slope value of *I*–*V* curving in HRS (Fig. 4).

## Conclusions

In summary, we investigated the p–n interfacial effects on the resistive switching and flexibility behavior of NiO/TiO_2_ heterojunction fabricated on a flexible PET substrate. The inherent merits, such as stored resistance states, ON/OFF ratio and endurance properties, were measured at different bending degrees. The resistive switching behavior is due to the formation and rupture of oxygen-vacancy conducting filaments at the p–n interface, which is modulated by electric-field-induced junction barriers. Compared to that of the unbent film, the ON/OFF ratio for bent samples exhibits slight improvement. Finite element studies and charge transport analysis indicate that the bending-induced micro-crack can facilitate the oxidation of the p–n interface and lead to the incomplete rupture of the conducting filaments.

## Conflicts of interest

There are no conflicts to declare.

## Supplementary Material

RA-008-C8RA01180J-s001
