# Supplementary material for: Bending effect on the resistive switching behavior of a NiO/TiO2 p–n heterojunction
Source: RSC Adv. 2018 May 30;8(35):19861–7. doi: 10.1039/c8ra01180j (PMC9080738; doi:10.1039/c8ra01180j)
Supplement: RA-008-C8RA01180J-s001 [file RA-008-C8RA01180J-s001.pdf]

Electronic Supplementary Material (ESI) for RSC Advances.
This journal is © The Royal Society of Chemistry 2018

Bending Effect on Resistive Switching behavior of NiO/TiO_2 p-n Heterojunction

Hai-peng Cui, Jian-chang Li,* and Hai-lin Yuan

Vacuum and Fluid Engineering Research Center, Northeastern University, Shenyang 110819, PR China

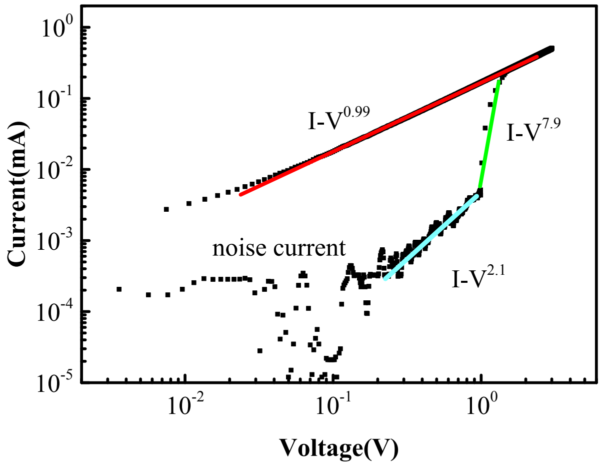

Figure. S1 Logarithmic plot and shop for HRS of I-V curves.

The I-$V^{0.99}$ fitting result demonstrates that electrons in the LRS are dominated by Ohmic conduction. However in the HRS, there contains three regimes.[1] At low voltage (<0.2 V), there is only some noise current of few pA, the injected electrons may be trapped by the film defects.[2] With bias increasing, the injected electrons become predominant, and a trap-filled-limited conduction is observed (I-$V^{2.1}$). When the traps are filled with injected electrons under high voltage, the device builds of the conductive path (I-$V^{7.9}$). Those results illustrate that the trap induced SCLC plays an important role in the HRS, as shown in Figure. S1.

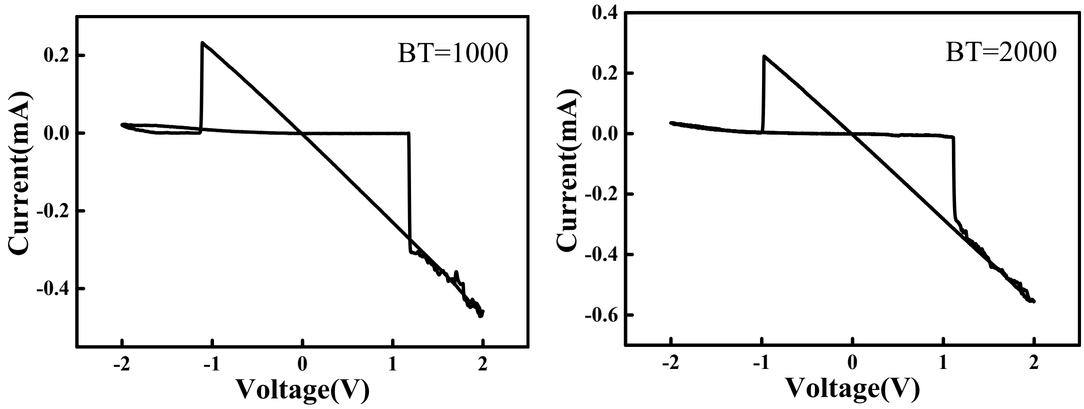

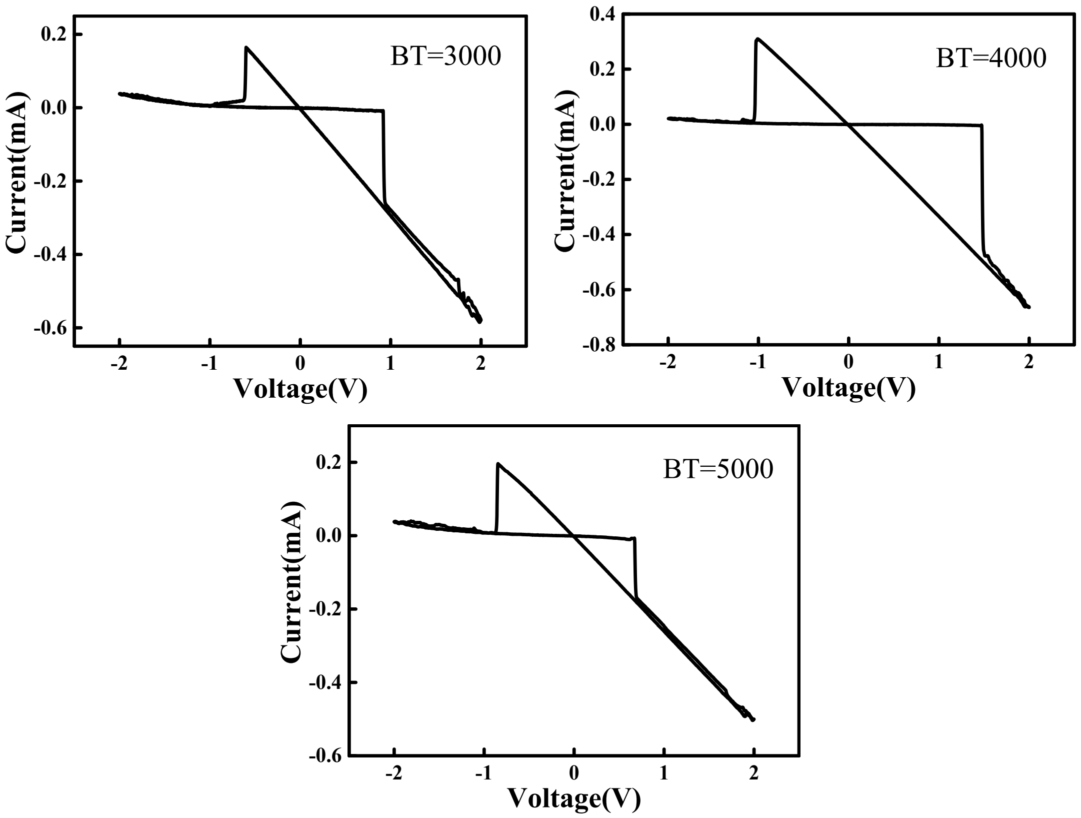

Figure. S2 Current-voltage data in different bending condition.

[1] G. D. Zhou, B. Sun, Y. Q. Yao, H. H. Zhang, A. K. Zhou, K. Alameh, B. F. Ding, and Q. L. Song, Appl. Phys. Lett. 2016, **109**, 143904.

[2] J. C. Li, X. Y. Hou, and Q. Cao, J. Alloys Compd. 2014, **115**, 164507.
